# Preditores de Qualidade de Vida, Ansiedade e Aceitação em Pacientes com Cardioversor-Desfibrilador Implantável

**DOI:** 10.36660/abc.20230590

**Published:** 2024-04-11

**Authors:** Laisa Arruda Silva, Katia Regina Silva, Sarah Caroline Martins Saucedo, Roberto Costa

**Affiliations:** 1 Hospital das Clínicas Faculdade de Medicina Universidade de São Paulo São Paulo SP Brasil Instituto do Coração do Hospital das Clínicas da Faculdade de Medicina da Universidade de São Paulo, São Paulo, SP – Brasil

**Keywords:** Desfibriladores Implantáveis, Qualidade de Vida, Ansiedade

## Abstract

**Fundamento:**

O cardioversor-desfibrilador implantável (CDI) pode causar níveis elevados de ansiedade e depressão, resultando em efeitos negativos na qualidade de vida.

**Objetivos:**

Avaliar a qualidade de vida, a ansiedade e a aceitação do CDI por meio de instrumentos de medida padronizados e identificar preditores de melhores respostas para cada um dos desfechos estudados.

**Método:**

Coorte prospectiva com pacientes submetidos a implante inicial de CDI ou reoperação para a manutenção do dispositivo. Os desfechos do estudo incluíram: qualidade de vida, ansiedade e aceitação do CDI. A mudança nos escores (30 e 180 dias) foi avaliada por meio da diferença mínima importante (DMI). Foi utilizada a análise univariada e o modelo de regressão logística multivariada para a identificação de preditores de melhores respostas, adotando-se o nível de significância de 5%.

**Resultados:**

De janeiro/2020 a junho/2021 foram incluídos 147 pacientes, com idade média de 55,3 ± 13,4 anos e predomínio do sexo masculino (72,1%). A DMI para qualidade de vida, a ansiedade e a aceitação do CDI foram observadas em 33 (22,4%), 36 (24,5%) e 43 (29,3%) pacientes, respectivamente. Idade igual ou maior que 60 anos (OR=2,5; IC 95%=1,14-5,53; p=0,022), ausência de fibrilação atrial (OR=3,8; IC 95%=1,26-11,63; p=0,017) e sexo feminino (OR=2,2; IC 95%=1,02-4,97; p=0,045) foram preditores independentes de melhores respostas para qualidade de vida, ansiedade e aceitação do CDI, respectivamente.

**Conclusão:**

A identificação de preditores para melhores escores de qualidade de vida, ansiedade e aceitação do dispositivo pode subsidiar a implementação de cuidados específicos para os pacientes com maiores chances de apresentar resultados desfavoráveis.

## Graphical abstract

**Figura Central:**
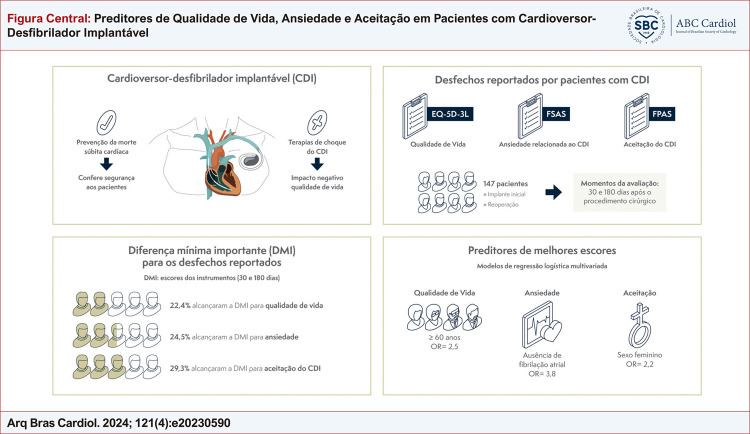
: Preditores de Qualidade de Vida, Ansiedade e Aceitação em Pacientes com Cardioversor-Desfibrilador Implantável

## Introdução

O cardioversor-desfibrilador implantável (CDI) é considerado uma das opções terapêuticas mais eficazes para a prevenção da morte súbita cardíaca (MSC) devido à sua capacidade de identificar e interromper arritmias ventriculares potencialmente fatais pela aplicação de terapias de choque.^1,2^ Embora esse dispositivo cardíaco salve vidas, os pacientes convivem com a sensação de que, a qualquer momento, podem receber uma terapia de choque.^3^ Por conta disso, pode ocorrer sofrimento psicológico, ansiedade, depressão e medo de falhas no funcionamento do dispositivo. Por outro lado, o CDI também confere segurança, por ser capaz de interromper as arritmias ventriculares letais, protegendo os pacientes contra os episódios imprevisíveis de MSC.^3,4^

No contexto atual, a avaliação de desfechos reportados pelos pacientes (do inglês, *patient reported outcomes*) tem se tornado uma ferramenta de grande relevância para a prática clínica. Essa abordagem mais abrangente visa incorporar métricas complementares aos resultados clínicos tradicionais tendo por base a perspectiva, as prioridades e as preferências dos pacientes.^5^ Nos últimos anos, vários estudos sobre o impacto do CDI nos desfechos reportados pelos pacientes foram publicados, no entanto, a grande heterogeneidade dos resultados tem impedido um melhor entendimento dos efeitos do CDI em termos de qualidade de vida, ansiedade e aceitação do dispositivo.^3,4,6^

A identificação de fatores que possam discriminar subgrupos com maior risco de apresentar desfechos psicossociais desfavoráveis ainda foi pouco explorada na literatura e os resultados variam consideravelmente entre os estudos. Até o presente momento, a ocorrência de terapias de choque do CDI,^7^ condições psicológicas pré-existentes como depressão,^8^ personalidade do tipo D^9^ e transtorno de ansiedade generalizada,^4^ sexo feminino,^10^ idade superior a 60 anos,^11^ a falta de suporte social^9^ e de conhecimento sobre a doença e o dispositivo^12^ foram os principais fatores associados ao impacto negativo do CDI sob a perspectiva dos pacientes.

Diante desse cenário, o objetivo do presente estudo foi avaliar os desfechos reportados por pacientes com CDI, incluindo a qualidade de vida relacionada à saúde, a ansiedade e a aceitação do dispositivo por meio de instrumentos de medida padronizados e identificar preditores de melhores respostas para cada um dos desfechos estudados.

## Métodos

### Desenho do estudo e aspectos éticos

Trata-se de um estudo de coorte prospectivo realizado em um hospital cardiológico da cidade de São Paulo. O estudo foi aprovado pelo Comitê de Ética em Pesquisa da Instituição. Todos os participantes assinaram o termo de consentimento livre e esclarecido (TCLE).

#### População estudada

Foram considerados elegíveis para o estudo os pacientes submetidos a procedimento cirúrgico de implante inicial de CDI ou reoperação para manutenção do dispositivo, com idade entre 18 e 90 anos. Não foram incluídos aqueles que apresentavam déficit cognitivo que pudesse comprometer o entendimento do conteúdo dos instrumentos de medida ou que não puderam ser contactados em tempo hábil para a aplicação do TCLE.

A amostra, definida por conveniência, foi composta por todos os pacientes que foram operados de maneira consecutiva no período do estudo e que atenderam aos critérios de elegibilidade.

## Etapas do estudo

### Seleção dos participantes da pesquisa

Os pacientes foram selecionados, de forma consecutiva, durante as visitas diárias que eram realizadas nas Unidades de Internação da nossa Instituição ou, eventualmente, por meio de contato telefônico após a alta hospitalar.

### Avaliação das características basais no momento da hospitalização índice

Após a inclusão no estudo, era realizado o levantamento do histórico clínico e das informações relacionadas à hospitalização índice do estudo, mediante consulta do prontuário e entrevista com os pacientes.

Nessa etapa, foram coletados os dados demográficos, o histórico de saúde, as condições clínicas atuais, as informações relacionadas ao episódio de internação hospitalar índice e os dados do procedimento cirúrgico.

A coleta de dados foi realizada em formulários eletrônicos desenvolvidos no software REDCap (*Research Electronic Data Capture*).^13^

### Avaliação dos desfechos reportados pelos pacientes

A avaliação dos desfechos reportados foi realizada em 30 e 180 dias após o procedimento cirúrgico pelo autopreenchimento dos instrumentos de medida, enviados aos participantes por WhatsApp ou e-mail, por entrevista telefônica ou presencial. Os instrumentos de medida utilizados foram o *EuroQol 5-dimensions, 3 levels* (EQ-5D-3L), o *Florida Shock Anxiety Scale* (FSAS) o *Florida Patient Acceptance Survey* (FPAS) para avaliar a qualidade de vida relacionada à saúde, o nível de ansiedade relacionado ao CDI e a aceitação do dispositivo, respectivamente.

O EQ-5D-3L é composto por um sistema descritivo que engloba cinco dimensões, (“mobilidade”, “autocuidado”, “atividades habituais”, “dor/desconforto” e “ansiedade/depressão”) com três níveis de gravidade (“sem problemas”, “alguns problemas” e “problemas extremos”). O estado de saúde é definido pela combinação dos níveis de cada uma das cinco dimensões, sendo representado por um número com cinco dígitos e totalizando 243 estados possíveis. Cada estado de saúde pode ser convertido em um escore único que incorpora as preferências sociais, variando de 0 (pior estado possível) a 1 (perfeita saúde). O instrumento também apresenta uma escala visual analógica para autoavaliação do estado de saúde que varia de 0 (pior) a 100 (melhor) pontos.^14^

O FSAS é o único instrumento específico disponível para avaliar o nível de ansiedade relacionado ao CDI e às terapias de choques.^6,15^Apresenta 10 perguntas com cinco opções de resposta (“nunca”, “quase nunca”, “de vez em quando”, “quase sempre” e “sempre”). O escore total do instrumento é determinado pela soma de todos os itens, podendo atingir 50 pontos, sendo que pontuações maiores refletem um nível mais elevado de ansiedade.^6,15^

O FPAS é o único instrumento específico disponível para avaliar o nível de aceitação do paciente em relação ao dispositivo cardíaco.^16,17^ É composto por 12 itens com opções de resposta dispostas em uma escala do tipo *Likert*, variando de 1 (“discordo totalmente”) a 5 (“concordo totalmente”). O escore total é determinado pela soma de todos os itens, transformada em uma escala de 0 (menor nível de aceitação) a 100 (maior nível de aceitação) pontos.^16,17^

## Desfechos do estudo

Os desfechos do estudo foram representados pelas alterações nos escores de qualidade de vida, ansiedade e aceitação do dispositivo quantificadas por meio da diferença mínima importante (DMI). Adotou-se um limiar de variação de 0,5 do desvio-padrão dos escores totais. Dessa forma, os pacientes que apresentaram alterações iguais ou superiores à metade do desvio-padrão do escore total foram considerados como tendo alcançado uma DMI para os construtos avaliados.

## Variáveis estudadas e análise estatística

Foram analisadas como variáveis independentes para os desfechos: dados demográficos, dados clínicos basais, dados do procedimento cirúrgico, dados da hospitalização índice e do seguimento clínico de 30 e 180 dias.

Foi realizada uma análise descritiva minuciosa utilizando medidas de tendência central (valores mínimos e máximos, médias, desvio-padrão e mediana) para as variáveis contínuas e o cálculo das frequências absolutas e relativas para as variáveis categóricas. O teste de Kolmogorov-Smirnov (KS) foi utilizado para a testagem da normalidade dos dados.

Para a comparação dos escores totais dos instrumentos, nos momentos de 30 e 180 dias, foi utilizado o teste t pareado. Para a comparação dos pacientes que atingiram ou não a DMI utilizou-se a análise univariada por meio dos testes “t” de Student não pareado, Exato de Fisher e Qui-quadrado, conforme a natureza dos dados. Com a finalidade de determinar os preditores de melhores respostas dos questionários foram desenvolvidos três modelos distintos (qualidade de vida, ansiedade e aceitação) de regressão logística multivariada pelo método de *stepwise*, sendo consideradas as variáveis que apresentaram p ≤ 0,10 na análise univariada. A magnitude do efeito das variáveis que constituíram o modelo final foi estimada pelo *odds ratio* (OR) e seus respectivos intervalos de confiança (IC) de 95%.

A análise estatística foi realizada no software *Statistical Package for the Social Sciences* (SPSS v. 17.0), adotando-se um nível de significância de 5%.

## Resultados

### Características basais dos pacientes

De janeiro de 2020 a junho de 2021, 258 pacientes foram submetidos a implante inicial ou à reoperação relacionada ao CDI. Destes, 147 atenderam aos critérios de elegibilidade e foram incluídos no estudo. Os motivos de não inclusão de 111 pacientes foram: recusa (25 pacientes), óbito hospitalar (10 pacientes), impossibilidade de preencher os questionários em tempo hábil (33 pacientes) e impossibilidade de contato para a aplicação do TCLE (43 pacientes).

As características demográficas, clínicas e as informações relacionadas ao CDI estão apresentadas na Tabela 1.

**Tabela 1:** – Características basais dos participantes da pesquisa

Características da amostra	N= 147
**Sexo, n (%)**	
Masculino	106 (72,1)
Feminino	41 (27,9)
**Idade (anos)**	
Média ± desvio padrão	55,3 ± 13,4
Variação	18,1 – 88,4
**Raça declarada, n (%)**	
Branca	122 (83,0)
Parda/mulato	14 (9,5)
Negra	9 (6,1)
Amarela	2 (1,4)
**Estado civil, n (%)**	
Solteiro	24 (16,3)
Casado	103 (70,1)
União estável	5 (3,4)
Divorciado/ Separado	8 (5,4)
Viúvo	7 (4,8)
**Escolaridade, n (%)**	
Ensino fundamental incompleto	41 (27,9)
Ensino fundamental completo	20 (13,6)
Ensino médio completo	54 (36,7)
Ensino superior	32 (21,8)
**Provedor de saúde, n (%)**	
Sistema único de saúde	133 (90,5)
Saúde suplementar	14 (9,5)
**Estado empregatício, n (%)**	
Aposentado	53 (36,2)
Emprego formal	44 (29,9)
Desempregado	19 (12,9)
Emprego informal	13 (8,8)
Informação não disponível	18 (12,2)
**Doença cardíaca estrutural, n (%)**	
Cardiopatia isquêmica	40 (27,2)
Cardiopatia não-isquêmica	40 (27,2)
Doença de Chagas	34 (23,1)
Cardiopatia hipertrófica	15 (10,2)
Canalopatias	10 (6,8)
Outras	8 (5,4)
**Comorbidades, n (%)**	
Insuficiência cardíaca	102 (69,4)
Parada cardíaca prévia/ taquicardia ventricular instável	100 (68,0)
Hipertensão arterial	69 (46,9)
Infarto do miocárdio prévio/ doença arterial coronariana	43 (29,3)
Fibrilação atrial	40 (27,2)
Diabetes mellitus	24 (16,3)
Insuficiência renal crônica	18 (12,2)
Valvopatias/ prótese valvares	15 (10,2)
Acidente vascular cerebral	15 (10,2)
Número total de comorbidades	9,4 ± 1,6
Uso de anticoagulante oral, n (%)	41 (27,9)
**Classe Funcional (New York Heart Association), n (%)**	
I – II	116 (78,9)
III – IV	31 (21,1)
**Procedimento cirúrgico realizado, n (%)**	
Implante inicial	67 (45,6)
Reoperação	80 (54,4)
**Indicação principal dos implantes iniciais, n (%)**	
Profilaxia secundária da morte súbita cardíaca	36 (24,5)
Profilaxia primária da morte súbita cardíaca	21 (14,3)
Ressincronização cardíaca + profilaxia da morte súbita	10 (6,8)
**Principal indicação dos procedimentos de reoperação, n (%)**	
Depleção natural do gerador de pulsos	39 (26,5)
Disfunção em cabos-eletrodos	25 (17,0)
Mudança no modo de estimulação	14 (9,5)
Depleção precoce do gerador de pulsos	1 (0,7)
Reimplante após remoção prévia do CDI por infecção	1 (0,7)
**Tipo de dispositivo, n (%)**	
CDI ventricular	31 (21,1)
CDI atrioventricular	77 (52,3)
CDI subcutâneo	2 (1,4)
Terapia de ressincronização cardíaca associada ao CDI	37 (25,2)

CDI: cardioversor-desfibrilador implantável.

### Qualidade de vida, ansiedade e aceitação do CDI

O tempo médio de seguimento foi de 6,2 ± 1,0 meses. Durante esse período, nenhum paciente faleceu ou foi perdido para seguimento. Apenas 6 (4,1%) indivíduos receberam terapias de choque do CDI.

Os escores totais do EQ-5D-3L para as avaliações realizadas em 30 e 180 dias foram 0,78 ± 0,21 e 0,76 ± 0,20 (p= 0,148), respectivamente. Quanto à percepção do estado geral de saúde, os escores médios foram 78,7 ± 18,4 e 73,8 ± 21,8 (p= 0,015), para as avaliações realizadas em 30 e 180 dias, respectivamente. Os domínios que apresentaram maiores taxas de problemas foram “ansiedade/ depressão” e “dor/ mal-estar” em ambas as avaliações (Tabela 2).

**Tabela 2:** – Distribuição das respostas dos participantes segundo os domínios do instrumento EQ-5D-3L, nas avaliações realizadas 30 e 180 dias após o procedimento cirúrgico

	Mobilidade		Cuidados Pessoais		Atividades habituais		Dor/ Mal-estar		Ansiedade/ Depressão	
				
Respostas	30 dias	180 dias	30 dias	180 dias	30 dias	180 dias	30 dias	180 dias	30 dias	180 dias
Sem problemas, n (%)	121 (82,3)	99 (67,3)	127 (86,4)	131 (89,1)	98 (66,7)	95 (64,6)	91 (61,9)	87 (59,2)	90 (61,2)	82 (55,8)
Alguns problemas, n (%)	25 (17,0)	48 (32,7)	19 (12,9)	15 (10,2)	41 (27,9)	44 (29,9)	52 (35,4)	59 (40,1)	50 (34,0)	61 (41,5)
Problemas importantes, n (%)	1 (0,7)	0 (0,0)	1 (0,7)	1 (0,7)	8 (5,4)	8 (5,4)	4 (2,7)	1 (0,7)	7 (4,8)	4 (2,7)
Qualquer problema*, n (%)	26 (17,7)	48 (32,7)	20 (13,6)	16 (10,9)	49 (33,3)	52 (35,3)	56 (38,1)	60 (40,8)	57 (38,8)	65 (44,2)
Valor de p	0,001		0,419		0,692		0,897		0,551	

*Qualquer problema = combinação dos problemas leves e importantes.

O escore total médio do instrumento FSAS, no geral, refletiu um estado de ansiedade leve na população, representado pela média de 23,5 ± 11,0 na avaliação de 30 dias e 23,9 ± 11,3 em 180 dias (p= 0,622). A análise dos itens do instrumento mostrou que mais de 30% dos pacientes assinalaram opções de resposta que denotam maior nível de ansiedade relacionada ao “medo de fazer exercícios físicos” e ao “medo de estar sozinho quando o dispositivo aplicar uma terapia de choque” (Tabela 3).

**Tabela 3:** – Distribuição das respostas dos participantes segundo os itens do instrumento FSAS, nas avaliações realizadas 30 e 180 dias após o procedimento cirúrgico

	Nunca		Quase nunca		De vez em quando		Quase sempre		Sempre		p
				
Itens do FSAS	30 dias	180 dias	30 dias	180 dias	30 dias	180 dias	30 dias	180 dias	30 dias	180 dias	
1 - Medo de fazer exercícios físicos	50 (34,0)	47 (32,0)	10 (6,8)	12 (8,2)	22 (15,0)	20 (13,6)	18 (12,2)	16 (10,9)	47 (32,0)	52 (35,4)	0,682
2 – Medo de estar sozinho quando receber o choque	70 (47,6)	66 (44,9)	13 (8,8)	11 (7,5)	18 (12,2)	17 (11,6)	9 (6,1)	13 (8,8)	37 (25,2)	40 (27,2)	0,408
3 – Medo de ficar nervoso/chateado	75 (51,0)	69 (46,9)	13 (8,8)	18 (12,2)	18 (12,2)	13 (8,8)	10 (6,8)	10 (6,8)	31 (21,1)	37 (25,2)	0,466
4 – Preocupação por não saber quando o CDI vai aplicar um choque	66 (44,9)	64 (43,5)	7 (4,8)	13 (8,8)	18 (12,2)	15 (10,2)	10 (6,8)	12 (8,2)	46 (31,3)	43 (29,3)	0,810
5 – Preocupação do CDI não funcionar	65 (44,2)	71 (48,3)	15 (10,2)	22 (15,0)	22 (15,0)	15 (10,2)	8 (5,4)	6 (4,1)	37 (25,2)	33 (22,4)	0,152
6 – Medo de tocar nas pessoas	112 (76,2)	111 (75,5)	9 (6,1)	6 (4,1)	6 (4,1)	7 (4,8)	4 (2,7)	6 (4,1)	16 (10,9)	17 (11,6)	0,488
7 – Preocupação em assustar as pessoas com o choque	76 (51,7)	80 (54,4)	12 (8,2)	11 (7,5)	15 (10,2)	17 (11,6)	10 (6,8)	9 (6,1)	34 (23,1)	30 (20,4)	0,428
8 – Preocupação de perceber o coração acelerado	68 (46,3)	64 (43,5)	11 (7,5)	19 (12,9)	21 (14,3)	15 (10,2)	12 (8,2)	9 (6,1)	35 (23,8)	40 (27,2)	0,603
9 – Pensar o tempo o todo em relação ao choque	85 (57,8)	75 (51,0)	15 (10,2)	23 (15,6)	19 (12,9)	17 (11,6)	5 (3,4)	8 (5,4)	23 (15,6)	24 (16,3)	0,467
10 – Evitar relações sexuais	113 (76,9)	106 (72,1)	7 (4,8)	5 (3,4)	12 (8,2)	18 (12,2)	4 (2,7)	9 (6,1)	11 (7,5)	9 (6,1)	0,210

CDI: cardioversor-desfibrilador implantável; FSAS: Florida Shock Anxiety Scale.

O escore total médio do FPAS foi 72,6 ± 16,1 e 74,7 ± 19,4 (p= 0,086) nos respectivos momentos de avaliação. A análise de cada item do instrumento mostrou que os pacientes apresentaram níveis de aceitação ao CDI próximos ao adequado, sendo que mais de 80% concordaram que “colocariam o aparelho novamente”, mais de 70% consideraram “o aparelho como melhor opção de tratamento” e mais de 60% responderam que “as vantagens do CDI são maiores que as desvantagens” (Tabela 4).

**Tabela 4:** – Distribuição das respostas dos participantes segundo os itens do instrumento FPAS, nas avaliações realizadas 30 e 180 dias após o procedimento cirúrgico

	Concordo totalmente		Concordo parcialmente		Nem concordo, nem discordo		Discordo parcialmente		Discordo totalmente		p
				
Itens do FPAS	30 dias	180 dias	30 dias	180 dias	30 dias	180 dias	30 dias	180 dias	30 dias	180 dias	
1 – Ficar deprimido ao pensar no aparelho	8 (5,14)	3 (2,0)	19 (12,9)	26 (17,7)	9 (6,1)	6 (4,1)	9 (6,1)	8 (5,4)	102 (69,4)	104 (70,7)	0,739
2 – Evitar fazer coisas que gosta	18 (12,2)	15 (10,2)	37 (25,2)	31 (21,1)	5 (3,4)	7 (4,8)	16 (10,9)	20 (13,6)	71 (48,3)	74 (50,3)	0,297
3 – Evitar atividades por sentir incômodo com aparelho	13 (8,8)	7 (4,8)	18 (12,2)	19 (12,9)	11 (7,5)	3 (2,0)	17 (11,6)	17 (11,6)	88 (59,9)	101 (68,7)	0,027
4 – Dificuldade em viver sem pensar no aparelho	18 (12,2)	13 (8,8)	25 (17,0)	22 (15,0)	6 (4,1)	5 (3,4)	16 (10,9)	16 (10,9)	82 (55,8)	91 (61,9)	0,138
5 – Considerar o aparelho como melhor opção de tratamento	128 (87,1)	115 (78,2)	11 (7,5)	19 (12,9)	5 (3,4)	8 (5,4)	3 (2,0)	2 (1,4)	0 (0,0)	3 (2,0)	0,021
6 – Certeza em condições de voltar a trabalhar	52 (35,4)	49 (33,3)	26 (17,7)	25 (17,0)	11 (7,5)	8 (5,4)	16 (10,9)	13 (8,8)	42 (28,6)	52 (35,4)	0,152
7 – Segurança em relação à doença	96 (65,3)	91 (61,9)	34 (23,1)	35 (23,8)	9 (6,1)	7 (4,8)	6 (4,1)	9 (6,1)	2 (1,4)	5 (3,4)	0,122
8 – Vantagens do CDI são maiores que as desvantagens	121 (82,3)	114 (77,6)	16 (10,9)	21 (14,3)	6 (4,1)	8 (5,4)	2 (1,4)	1 (0,7)	2 (1,4)	3 (2,0)	0,340
9 – Colocar o aparelho novamente	124 (84,4)	125 (85,0)	12 (8,2)	14 (9,5)	7 (4,8)	4 (2,7)	1 (0,7)	2 (1,4)	3 (2,0)	2 (1,4)	0,655
10 – Vida normal	41 (27,9)	57 (38,8)	64 (43,5)	49 (33,3)	6 (4,1)	4 (2,7)	23 (15,6)	18 (12,2)	13 (8,8)	19 (12,9)	0,628
11 – Fazer as coisas para a família	28 (19,0)	19 (12,9)	41 (27,9)	43 (29,3)	6 (4,1)	6 (4,1)	22 (15,0)	20 (13,6)	50 (34,0)	59 (40,1)	0,133
12 – Preocupação em fazer atividades físicas	48 (32,7)	33 (22,4)	49 (33,3)	44 (29,9)	7 (4,8)	4 (2,7)	11 (7,5)	16 (10,9)	32 (21,8)	50 (34,0)	0,002

CDI: cardioversor-desfibrilador implantável; FPAS: Florida Patient Acceptance Survey

### Preditores de qualidade de vida, ansiedade e aceitação do CDI

Diferença mínima importante para os construtos qualidade de vida, ansiedade e aceitação do CDI foi observada em 33 (22,4%), 36 (24,5%) e 43 (29,3%) pacientes, respectivamente (Tabela 5). A comparação dos grupos de pacientes que alcançaram ou não a DMI para cada um dos desfechos estudados, permitiu identificar que os grupos apresentaram características similares em relação a maioria das variáveis estudadas, sendo possível observar que não houve diferenças significativas entre os pacientes submetidos a implante inicial do CDI ou reoperação, conforme detalhado no Material Suplementar (Tabela S1).

**Tabela 5:** – Diferença mínima importante para os desfechos estudados obtida pela comparação das avaliações realizadas em 30 e 180 dias após o procedimento cirúrgico

	Qualidade de vida (EQ-5D-3L)	Ansiedade relacionada ao CDI (FSAS)	Aceitação do CDI (FPAS)
**Taxa de indivíduos que não alcançaram a DMI, n (%)**	114 (77,6%)	111 (75,5%)	104 (70,7%)
**Taxa de indivíduos que alcançaram a DMI, n (%)**	33 (22,4%)	36 (24,5%)	43 (29,3%)
**Valor da DMI alcançada**	> 0,102	> 5,48	> 6,66
**Escores dos indivíduos que alcançaram a DMI, média ± desvio-padrão**	0,78 ± 0,2	23,5 ± 10,9	72,6 ± 16,7

CDI: cardioversor-desfibrilador implantável; DMI: diferença mínima importante; EQ-5D-3L: EuroQoL 5-dimensions, 3 levels; FPAS: Florida Patient Acceptance Survey; FSAS: Florida Shock Anxiety Scale.

Idade igual ou maior que 60 anos (OR=2,5; IC 95%=1,14-5,53; p=0,022), ausência de fibrilação atrial (OR=3,8; IC 95%=1,26-11,63; p=0,017) e sexo feminino (OR=2,2; IC 95%=1,02-4,97; p=0,045) foram preditores independentes de melhores respostas para qualidade de vida, ansiedade e aceitação do CDI, respectivamente.

## Discussão

Os desfechos reportados por pacientes são importantes indicadores de saúde, relatados a partir da perspectiva do próprio indivíduo a respeito de sua doença e tratamento, contribuindo para uma abordagem centrada no paciente.^5,18^Apesar da segurança que o CDI proporciona, os indivíduos podem evoluir com comprometimento na qualidade de vida, principalmente em decorrência das terapias de choque.^3,4,7^ Considerando a necessidade do melhor entendimento do impacto do CDI, este estudo avaliou a qualidade de vida, a ansiedade e a aceitação, utilizando, pela primeira vez no Brasil, os instrumentos de medida EQ-5D-3L, FSAS e FPAS.

Nosso estudo reflete dados da prática clínica real de um hospital cardiológico terciário, principalmente por incluir todos os pacientes operados em um determinado período, independentemente do perfil clínico, do procedimento realizado e do tipo de CDI. Encontramos uma proporção semelhante de pacientes com cardiopatia isquêmica (27,2%), não isquêmica (27,2%) e Doença de Chagas (23,1%) e houve maior representatividade dos procedimentos de reoperação (54,4%).

Na avaliação da qualidade de vida, os resultados do estado geral de saúde, obtidos pela escala visual analógica (EQ-EVA), apresentaram médias com variação de 73,8 a 78,7, semelhante a valores reportados na literatura, que variaram de 62,4 a 77,6, de acordo com o momento do estudo e a indicação do CDI.^19,20^ Para o construto ansiedade relacionada ao CDI, os escores médios obtidos variaram de 23,5 a 23,9, denotando um nível leve de ansiedade, à semelhança de um estudo transversal que reportou escores médios de 22,1 pontos.^8^ Para a aceitação do dispositivo, os escores obtidos de 72,6 a 74,7, em 30 e 180 dias, respectivamente, mostraram um nível adequado de aceitação, semelhante a estudos prévios, que reportaram pontuações que variaram de 64,7 a 66,5.^8,21^

A taxa de pacientes que alcançaram a DMI para os construtos qualidade de vida, ansiedade e aceitação do CDI foi de 22,4%, 24,5% e 29,3%, respectivamente. Na literatura, a DMI tem sido adotada por refletir resultados que vão além dos valores de significância estatística, permitindo detectar mudanças nos escores dos instrumentos de medida que podem representar alterações significativas do ponto de vista dos pacientes.^5,22^ Um estudo que utilizou a DMI para classificar os indivíduos submetidos a implante de marca-passo em relação à melhora dos escores de qualidade de vida identificou taxas de 30% a 59% de pacientes que alcançaram a DMI.^23^ Embora o marca-passo seja um dispositivo muito semelhante ao CDI, por atuar na correção da frequência cardíaca, promove um benefício hemodinâmico perceptível, de tal forma que, habitualmente, os pacientes relatam melhorias em seus sintomas. Em contrapartida, os pacientes com CDI geralmente não percebem nenhum benefício clínico após o implante do dispositivo e ainda convivem com a possibilidade de receberem terapias de choque.^3^

Não obstante o estudo não tenha sido desenhado especificamente para comparar os desfechos entre os diferentes tipos de procedimentos, observou-se uma proporção semelhante de pacientes que alcançaram a DMI para os três construtos analisados, tanto entre os indivíduos que tinham o CDI previamente implantado, como naqueles que foram submetidos a implante inicial. Esses achados sugerem que o tipo de procedimento, implantes iniciais ou reoperações, não teve impacto nos resultados do estudo, de forma semelhante ao que tem sido reportado na literatura.^7-12^

A idade, maior ou igual a 60 anos, foi considerada um preditor de qualidade de vida, aumentando em 2,5 vezes as chances de obter melhores escores. Condizente com estes resultados, pacientes com idade média de 64,7 ± 9,4 anos de uma coorte apresentaram melhores escores de qualidade de vida.^24^ Melhor qualidade de vida nessa faixa etária poderia ser explicada principalmente pela aceitação do dispositivo, pois, comparado a indivíduos jovens, já foi demonstrado que estes apresentam maiores dificuldades na aceitação do CDI.^24,25^ Em contrapartida, a idade acima de 60 anos também foi identificada como preditor para o comprometimento na qualidade de vida, justificada pela percepção de pior estado de saúde, preocupações e mudanças no estilo de vida dos pacientes que se encontram nessa faixa etária.^11^

A ausência de fibrilação atrial foi identificada como preditor de melhores escores para ansiedade, aumentando em 3,8 vezes a chance de melhores respostas. Dos pacientes com CDI, aproximadamente 25% apresentam fibrilação atrial^26^ e a ansiedade nesses indivíduos poderia ser explicada pela preocupação com as palpitações e com o uso de anticoagulantes, por conta do risco efeitos adversos, como sangramento e tromboembolismo.^27^ Dessa forma, é justificável que, neste estudo, os pacientes que tiveram melhores escores para ansiedade não tinham o diagnóstico de fibrilação atrial.

Para a aceitação do CDI, o sexo feminino foi identificado como preditor de melhores escores, aumentando em 2,2 vezes a chance de melhores respostas. Apesar de altos níveis de aceitação do CDI terem sido relatados na população feminina,^28^ também foi demonstrado que, em razão da influência na imagem corporal, as mulheres podem apresentar dificuldades de aceitação do dispositivo.^29^

Embora este estudo tenha permitido identificar preditores de melhores escores em desfechos reportados por pacientes com CDI, a generalização dos resultados é limitada, principalmente pelo fato de que a casuística foi reduzida e de que os dados refletiram a realidade de um único serviço. A realização de novos estudos com casuística mais representativa e abrangência multicêntrica poderá contribuir para a confirmação dos resultados, assim como para a identificação de outros preditores.

## Conclusões

O presente estudo permitiu conhecer melhor o perfil dos pacientes com CDI, em termos de qualidade de vida, ansiedade e aceitação do dispositivo e demonstrou a estabilidade dos escores obtidos entre os dois momentos de avaliação. A identificação de preditores para melhores escores de qualidade de vida, ansiedade e aceitação do dispositivo pode subsidiar a implementação de cuidados específicos para os pacientes com maiores chances de apresentar resultados desfavoráveis.
