# Supplementary material for: Preditores de Qualidade de Vida, Ansiedade e Aceitação em Pacientes com Cardioversor-Desfibrilador Implantável
Source: Arq Bras Cardiol. 2024 Apr 11;121(4):e20230590. [Article in Portuguese] doi: 10.36660/abc.20230590 (PMC11098579; doi:10.36660/abc.20230590)
Supplement: Translation

# Translation: Predictors of Quality of Life, Anxiety and Acceptance in Patients with Implantable Cardioverter-Defibrillator

**DOI:** 10.36660/abc.20230590i

**Authors:** Laisa Arruda Silva, Katia Regina Silva, Sarah Caroline Martins Saucedo, Roberto Costa

**Affiliations:** 1 Brazil Instituto do Coração do Hospital das Clínicas da Faculdade de Medicina da Universidade de São Paulo, São Paulo, SP – Brazil

**Keywords:** Implantable Defibrillators, Quality of Life, Anxiety

## Abstract

**Background:**

An implantable cardioverter-defibrillator (ICD) can cause high levels of anxiety and depression, resulting in negative effects on quality of life.

**Objectives:**

To evaluate the quality of life, anxiety, and acceptance of the ICD using standardized measurement instruments and identify predictors of better responses for each of the outcomes studied.

**Method:**

This is a prospective cohort study with patients undergoing initial ICD implantation or reoperation to maintain the device. The study outcomes included quality of life, anxiety, and acceptance of the ICD. The change in scores (30 and 180 days) was assessed using the minimal important difference (MID). Univariate analysis and the multivariate logistic regression model were used to identify predictors of better responses, adopting a significance level of 5%.

**Results:**

A total of 147 patients were included between January/2020 to June/2021, with a mean age of 55.3 ± 13.4 years and a predominance of males (72.1%). The MID for quality of life, anxiety, and ICD acceptance were observed in 33 (22.4%), 36 (24.5%) and 43 (29.3%) patients, respectively. Age equal to or greater than 60 years (OR=2.5; 95%CI=1.14-5.53; p=0.022), absence of atrial fibrillation (OR=3.8; 95%CI=1.26-11.63; p=0.017) and female gender (OR=2.2; 95%CI=1.02-4.97; p=0.045) were independent predictors of better responses to quality of life, anxiety and acceptance of the ICD, respectively.

**Conclusion:**

The identification of predictors for better quality of life scores, anxiety, and acceptance of the device can support the implementation of specific care for patients with a greater chance of presenting unfavorable results.

## Introduction

The implantable cardioverter-defibrillator (ICD) is considered one of the most effective therapeutic options for preventing sudden cardiac death (SCD) due to its ability to identify and interrupt potentially fatal ventricular arrhythmias through the application of shock therapies.^1,2^ Although this cardiac device saves lives, patients live with the feeling that they could receive shock therapy at any moment.^3^ In turn, psychological suffering, anxiety, depression, and fear of device failures may occur. On the other hand, the ICD also provides safety, as it is capable of interrupting lethal ventricular arrhythmias, protecting patients against unpredictable episodes of SCD.^3,4^

In the current context, evaluation of patient-reported outcomes (PROs) has become a highly relevant tool for clinical practice. This more comprehensive approach aims to incorporate complementary metrics to traditional clinical outcomes based on patients’ perspectives, priorities, and preferences.^5^ Several studies on the impact of ICD on PROs have been published in recent years; however, great heterogeneity of results has prevented a better understanding of the effects of the ICD in terms of quality of life, anxiety, and acceptance of the device.^3,4,6^

Identifying factors that may discriminate subgroups at greater risk of presenting unfavorable psychosocial outcomes has still been little explored in the literature and results vary considerably between studies. To date, the occurrence of ICD shock therapies^7^, pre-existing psychological conditions such as depression,^8^ type D^9^ personality and generalized anxiety disorder,^4^ female sex,^10^ age over 60 years,^11^ lack of social support,^9^ and knowledge about the disease and the device^12^ were the main factors associated with the negative impact of the ICD from the patients’ perspective.

Given this scenario, the objective of the present study was to evaluate PROs in adults with ICD, including health-related quality of life, anxiety, and acceptance of the device using standardized measurement instruments, and identify predictors of better responses for each one of the outcomes studied.

## Methods

### Study design and ethical aspects

This is a prospective cohort study conducted in a cardiology hospital in the city of São Paulo, Brazil. The Institution’s Research Ethics Committee approved the study. All participants signed the informed consent form.

### Study population

Patients who underwent a surgical procedure for initial ICD implantation or reoperation, aged between 18 and 90 years, were considered eligible for the study. Those who had a cognitive deficit that could compromise understanding the content of the measurement instruments or who could not be contacted in a timely manner to apply the informed consent form were not included.

The sample was defined by convenience, being composed of all patients who underwent consecutive surgery during the study period and who met the eligibility criteria.

### Study stages

#### Study participant selection

Patients were consecutively selected during daily visits, which were performed in the Inpatient Units of our Institution, or eventually through telephone contact after hospital discharge.

#### Assessment of baseline characteristics at the index hospitalization

After inclusion in the study, the clinical history and information related to the hospitalization index of the study were collected by consulting the medical records and interviewing the patients. At this stage, demographic data, health history, current clinical conditions, information related to the index hospital episode, and data on the surgical procedure were collected. Data were collected using electronic forms developed in the REDCap software (Research Electronic Data Capture).^13^

#### Assessment of patient-reported outcomes

The PRO evaluation was performed 30 and 180 days after the surgical procedure by self-completing the measurement instruments sent to participants via WhatsApp, e-mail, telephone, or in-person interviews. The measuring instruments used were the EuroQol 5-dimensions, 3 levels (EQ-5D-3L), the Florida Shock Anxiety Scale (FSAS), and the Florida Patient Acceptance Survey (FPAS) to assess health-related quality of life, the anxiety level related to the ICD and acceptance of the device, respectively.

The EQ-5D-3L descriptive system comprises five dimensions (“mobility”, “self-care”, “usual activities”, “pain/discomfort” and “anxiety/depression”) with three levels of severity (“no problems”, “some problems”, and “extreme problems”). Health status is defined by combining the levels of each of the five dimensions, represented by a five-digit number and totaling 243 possible states. Each health state can be converted into a unique score that incorporates social preferences, ranging from 0 (worst possible state) to 1 (perfect health). The instrument also presents a visual analogue scale for self-assessment of health status that ranges from 0 (worst) to 100 (best) points.^14^

The FSAS is the only specific instrument available to assess the anxiety level related to the ICD and shock therapies.^6,15^ It presents 10 questions with five answer options (“never”, “almost never”, “occasionally”, “almost always,” and “always”). The instrument’s total score is determined by the sum of all items, reaching 50 points, with higher scores reflecting a higher anxiety level. ^6,15^

The FPAS is the only specific instrument available to assess the patient’s acceptance level in relation to the cardiac device.^16,17^ It consists of 12 items with response options arranged on a Likert-type scale, ranging from 1 (“totally disagree”) to 5 (“totally agree”). The sum of all items determines the total score, transformed into a scale from 0 (lowest acceptance level) to 100 (highest acceptance level) points.^16,17^

## Study outcomes

The study outcomes were represented by changes in quality of life, anxiety and device acceptance scores quantified using the minimal important difference (MID). A variation threshold of 0.5 of the standard deviation of total scores was adopted. Therefore, patients who presented changes equal to or greater than half the standard deviation of the total score were considered to have achieved a MID for the constructs assessed.

## Studied variables and statistical analysis

The following were analyzed as independent variables for outcomes: demographic data, baseline clinical data, data from the surgical procedure, data from the hospitalization index and clinical follow-up of 30 and 180 days.

A detailed descriptive analysis was conducted using measures of central tendency (minimum and maximum values, means, standard deviation, and median) for continuous variables and the calculation of absolute and relative frequencies for categorical variables. The Kolmogorov-Smirnov test (KS) was employed to assess the normality of the data.

The paired t-test was used to compare the total scores of the instruments at 30 and 180 days. To compare patients who achieved or did not achieve the DMI, univariate analysis was conducted using the unpaired Student’s t-test, Fisher’s Exact test, and Chi-square test, depending on the nature of the data. In order to determine the predictors of better responses to the questionnaires, three different models (quality of life, anxiety, and acceptance) of multivariate logistic regression were developed using the stepwise method, considering the variables that presented p ≤ 0.10 in the univariate analysis. The effect magnitude of the variables that constituted the final model was estimated by the odds ratio (OR) and their respective 95% confidence intervals (CI). Statistical analysis was performed using the Statistical Package for the Social Sciences (SPSS v. 17.0) software program, adopting a significance level of 5%.

## Results

### Baseline characteristics

A total of 258 patients underwent initial ICD implantation or ICD-related reoperation from January 2020 to June 2021. Of these, 147 met the eligibility criteria and were included in the study. The reasons for not including 111 patients were refusal (25 patients), hospital death (10 patients), impossibility of completing the questionnaires in a timely manner (33 patients), and impossibility of contact to apply the informed consent form (43 patients). The demographic and clinical characteristics, along with information related to the ICD, are presented in Table 1.

**Table 1:** – Baseline characteristics of research participants

Sample characteristics	N= 147
**Sex, n (%)**	
Male	106 (72.1)
Female	41 (27.9)
**Age (years)**	
Mean ± standard deviation	55.3 ± 13.4
Variation	18.1 – 88.4
**Declared race, n (%)**	
White	122 (83.0)
Brown/mulatto	14 (9.5)
Black	9 (6.1)
Yellow	2 (1.4)
**Marital status, n (%)**	
Single	24 (16.3)
Married	103 (70.1)
Stable union	5 (3.4)
Divorced/Separated	8 (5.4)
Widow	7 (4.8)
**Education, n (%)**	
Incomplete elementary	41 (27.9)
Completed elementary	20 (13.6)
Completed high school	54 (36.7)
Post-secondary	32 (21.8)
**Health provider, n (%)**	
Unified Health System (public)	133 (90.5)
Health insurance	14 (9.5)
**Employment status, n (%)**	
Retired	53 (36.2)
Formal employment	44 (29.9)
Unemployed	19 (12.9)
Informal employment	13 (8.8)
Information not available	18 (12.2)
**Structural heart disease, n (%)**	
Ischemic heart disease	40 (27.2)
Non-ischemic heart disease	40 (27.2)
Chagas disease	34 (23.1)
Hypertrophic heart disease	15 (10.2)
Channelopathies	10 (6.8)
Others	8 (5.4)
**Comorbidities, n (%)**	
Heart failure	102 (69.4)
Previous cardiac arrest/unstable ventricular tachycardia	100 (68.0)
Arterial hypertension	69 (46.9)
Previous myocardial infarction/coronary artery disease	43 (29.3)
Atrial fibrillation	40 (27.2)
Diabetes mellitus	24 (16.3)
Chronic kidney failure	18 (12.2)
Valvular heart disease/ valve prosthesis	15 (10.2)
Stroke	15 (10.2)
Total number of comorbidities	9.4 ± 1.6
Use of oral anticoagulants, n (%)	41 (27.9)
**Functional Class (New York Heart Association), n (%)**	
I – II	116 (78.9)
III – IV	31 (21.1)
**Surgical procedure performed, n (%)**	
Initial implant	67 (45.6)
Reoperation	80 (54.4)
**Main indication of initial implants, n (%)**	
Secondary prophylaxis of sudden cardiac death	36 (24.5)
Primary prophylaxis of sudden cardiac death	21 (14.3)
Cardiac resynchronization + sudden death prophylaxis	10 (6.8)
**Main indication of reoperation procedures, n (%)**	
Natural depletion of the pulse generator	39 (26.5)
Lead dysfunction	25 (17.0)
Upgrade procedure	14 (9.5)
Early pulse generator depletion	1 (0.7)
Reimplantation after previous ICD removal due to infection	1 (0.7)
**Device type, n (%)**	
Ventricular ICD	31 (21.1)
Atrioventricular ICD	77 (52.3)
Subcutaneous ICD	2 (1.4)
Cardiac resynchronization therapy associated with ICD	37 (25.2)

ICD: implantable cardioverter-defibrillator.

### Quality of life, anxiety, and ICD acceptance

The average follow-up time was 6.2 ± 1.0 months. During this period, no patient died or was lost to follow-up. Only 6 (4.1%) subjects received ICD shock therapies.

The total EQ-5D-3L scores for the assessments performed at 30 and 180 days were 0.78 ± 0.21 and 0.76 ± 0.20 (p=0.148), respectively. The average scores regarding the perception of general health status were 78.7 ± 18.4 and 73.8 ± 21.8 (p=0.015) for the assessments carried out in 30 and 180 days, respectively. The domains that presented the highest rates of problems were “anxiety/depression” and “pain/discomfort” in both assessments (Table 2).

**Table 2:** – Distribution of participants’ responses according to the domains of the EQ-5D-3L instrument in assessments carried out 30 and 180 days after the surgical procedure

	Mobility		Self-care		Usual activities		Pain/Discomfort		Anxiety/ Depression	
				
Responses	30 days	180 days	30 days	180 days	30 days	180 days	30 days	180 days	30 days	180 days
No problems, n (%)	121 (82.3)	99 (67.3)	127 (86.4)	131 (89.1)	98 (66.7)	95 (64.6)	91 (61.9)	87 (59.2)	90 (61.2)	82 (55.8)
Minor problems, n (%)	25 (17.0)	48 (32.7)	19 (12.9)	15 (10.2)	41 (27.9)	44 (29.9)	52 (35.4)	59 (40.1)	50 (34.0)	61 (41.5)
Major problems, n (%)	1 (0.7)	0 (0.0)	1 (0.7)	1 (0.7)	8 (5.4)	8 (5.4)	4 (2.7)	1 (0.7)	7 (4.8)	4 (2.7)
Multiple problems*, n (%)	26 (17.7)	48 (32.7)	20 (13.6)	16 (10.9)	49 (33.3)	52 (35.3)	56 (38.1)	60 (40.8)	57 (38.8)	65 (44.2)
p value	0.001		0.419		0.692		0.897		0.551	

*Multiple problems = combination of minor and major problems

The mean total score of the FSAS instrument reflected a state of mild anxiety in the population, represented by an average of 23.5 ± 11.0 in the 30-day assessment and 23.9 ± 11.3 in 180 days (p= 0.622). Analysis of the instrument’s items showed that more than 30% of patients marked response options that denote a higher anxiety level related to “being scared to exercise” and “being alone when the ICD fires and I need help.” (Table 3).

**Table 3:** – Distribution of participants’ responses according to the items of the FSAS instrument in assessments carried out 30 and 180 days after the surgical procedure

	Never		Almost never		Sometimes		Almost always		Always		p
				
FSAS items	30 days	180 days	30 days	180 days	30 days	180 days	30 days	180 days	30 days	180 days	
1 - Fear of doing physical exercise	50 (34.0)	47 (32.0)	10 (6.8)	12 (8.2)	22 (15.0)	20 (13.6)	18 (12.2)	16 (10.9)	47 (32.0)	52 (35.4)	0.682
2 – Fear of being alone when receiving the shock	70 (47.6)	66 (44.9)	13 (8.8)	11 (7.5)	18 (12.2)	17 (11.6)	9 (6.1)	13 (8.8)	37 (25.2)	40 (27.2)	0.408
3 – Fear of getting nervous/upset	75 (51.0)	69 (46.9)	13 (8.8)	18 (12.2)	18 (12.2)	13 (8.8)	10 (6.8)	10 (6.8)	31 (21.1)	37 (25.2)	0.466
4 – Concern about not knowing when the ICD will deliver a shock	66 (44.9)	64 (43.5)	7 (4.8)	13 (8.8)	18 (12.2)	15 (10.2)	10 (6.8)	12 (8.2)	46 (31.3)	43 (29.3)	0.810
5 – Concern about the ICD not working	65 (44.2)	71 (48.3)	15 (10.2)	22 (15.0)	22 (15.0)	15 (10.2)	8 (5.4)	6 (4.1)	37 (25.2)	33 (22.4)	0.152
6 – Fear of touching people	112 (76.2)	111 (75.5)	9 (6.1)	6 (4.1)	6 (4.1)	7 (4.8)	4 (2.7)	6 (4.1)	16 (10.9)	17 (11.6)	0.488
7 – Concern about scaring people with shock	76 (51.7)	80 (54.4)	12 (8.2)	11 (7.5)	15 (10.2)	17 (11.6)	10 (6.8)	9 (6.1)	34 (23.1)	30 (20.4)	0.428
8 – Concern about noticing their heart racing	68 (46.3)	64 (43.5)	11 (7.5)	19 (12.9)	21 (14.3)	15 (10.2)	12 (8.2)	9 (6.1)	35 (23.8)	40 (27.2)	0.603
9 – Thinking about the shock all the time	85 (57.8)	75 (51.0)	15 (10.2)	23 (15.6)	19 (12.9)	17 (11.6)	5 (3.4)	8 (5.4)	23 (15.6)	24 (16.3)	0.467
10 – Avoid sexual relations	113 (76.9)	106 (72.1)	7 (4.8)	5 (3.4)	12 (8.2)	18 (12.2)	4 (2.7)	9 (6.1)	11 (7.5)	9 (6.1)	0.210

ICD: implantable cardioverter-defibrillator; FSAS: Florida Shock Anxiety Scale.

The mean total FPAS score was 72.6 ± 16.1 and 74.7 ± 19.4 (p= 0.086) at the respective assessment moments. The analysis of each item of the instrument showed that patients presented ICD acceptance levels close to adequate, with more than 80% agreeing that they would “receive the device again”, more than 70% considering “the device was the best treatment option”, and more than 60% responded that “the positive benefits of this device out-weigh the negatives” (Table 4).

**Table 4:** – Distribution of participants’ responses according to the items of the FPAS instrument in assessments carried out 30 and 180 days after the surgical procedure

	Completely agree		Partially agree		Neither agree nor disagree		Partially disagree		Completely disagree		p
				
FPAS items	30 days	180 days	30 days	180 days	30 days	180 days	30 days	180 days	30 days	180 days	
1 – Feeling depressed when thinking about the device	8 (5.14)	3 (2.0)	19 (12.9)	26 (17.7)	9 (6.1)	6 (4.1)	9 (6.1)	8 (5.4)	102 (69.4)	104 (70.7)	0.739
2 – Avoid doing things you like	18 (12.2)	15 (10.2)	37 (25.2)	31 (21.1)	5 (3.4)	7 (4.8)	16 (10.9)	20 (13.6)	71 (48.3)	74 (50.3)	0.297
3 – Avoid activities because you feel uncomfortable with braces	13 (8.8)	7 (4.8)	18 (12.2)	19 (12.9)	11 (7.5)	3 (2.0)	17 (11.6)	17 (11.6)	88 (59.9)	101 (68.7)	0.027
4 – Difficulty living without thinking about the device	18 (12.2)	13 (8.8)	25 (17.0)	22 (15.0)	6 (4.1)	5 (3.4)	16 (10.9)	16 (10.9)	82 (55.8)	91 (61.9)	0.138
5 – Consider the device as the best treatment option	128 (87.1)	115 (78.2)	11 (7.5)	19 (12.9)	5 (3.4)	8 (5.4)	3 (2.0)	2 (1.4)	0 (0.0)	3 (2.0)	0.021
6 – Certainty of being able to return to work	52 (35.4)	49 (33.3)	26 (17.7)	25 (17.0)	11 (7.5)	8 (5.4)	16 (10.9)	13 (8.8)	42 (28.6)	52 (35.4)	0.152
7 – Safety in relation to disease	96 (65.3)	91 (61.9)	34 (23.1)	35 (23.8)	9 (6.1)	7 (4.8)	6 (4.1)	9 (6.1)	2 (1.4)	5 (3.4)	0.122
8 – The advantages of the ICD are greater than the disadvantages	121 (82.3)	114 (77.6)	16 (10.9)	21 (14.3)	6 (4.1)	8 (5.4)	2 (1.4)	1 (0.7)	2 (1.4)	3 (2.0)	0.340
9 – Put the device back on	124 (84.4)	125 (85.0)	12 (8.2)	14 (9.5)	7 (4.8)	4 (2.7)	1 (0.7)	2 (1.4)	3 (2.0)	2 (1.4)	0.655
10 – Normal life	41 (27.9)	57 (38.8)	64 (43.5)	49 (33.3)	6 (4.1)	4 (2.7)	23 (15.6)	18 (12.2)	13 (8.8)	19 (12.9)	0.628
11 – Doing things for the family	28 (19.0)	19 (12.9)	41 (27.9)	43 (29.3)	6 (4.1)	6 (4.1)	22 (15.0)	20 (13.6)	50 (34.0)	59 (40.1)	0.133
12 – Concern about doing physical activities	48 (32.7)	33 (22.4)	49 (33.3)	44 (29.9)	7 (4.8)	4 (2.7)	11 (7.5)	16 (10.9)	32 (21.8)	50 (34.0)	0.002

ICD: implantable cardioverter-defibrillator; FPAS: Florida Patient Acceptance Survey.

### Predictors of quality of life, anxiety, and ICD acceptance

Minimal important difference in the construct’s quality of life, anxiety, and ICD acceptance was observed in 33 (22.4%), 36 (24.5%), and 43 (29.3%) patients, respectively (Table 5). The comparison of patient groups who achieved or did not achieve DMI for each of the studied outcomes allowed the identification that the groups exhibited similar characteristics concerning most of the studied variables. It is noteworthy that no significant differences were observed between patients undergoing the initial ICD implantation or reoperation, as detailed in the Supplementary Material (Table S1).

**Table 5:** – Minimal important difference for the studied outcomes obtained by comparing assessments carried out 30 and 180 days after the surgical procedure

	Quality of life (EQ-5D-3L)	Anxiety related to the ICD (FSAS)	Device acceptance (FPAS)
**Rate of individuals who did not achieve MID, n (%)**	114 (77.6%)	111 (75.5%)	104 (70.7%)
**Rate of individuals who achieved MID, n (%)**	33 (22.4%)	36 (24.5%)	43 (29.3%)
**MID value achieved**	> 0.102	> 5.48	> 6.66
**Scores of individuals who achieved MID, mean ± standard deviation**	0.78 ± 0.2	23.5 ± 10.9	72.6 ± 16.7

ICD: implantable cardioverter-defibrillator; MID: minimal important difference; EQ-5D-3L: EuroQoL 5-dimensions, 3 levels; FPAS: Florida Patient Acceptance Survey; FSAS: Florida Shock Anxiety Scale.

Age equal to or greater than 60 years (OR=2.5; 95% CI=1.14-5.53; p=0.022), absence of atrial fibrillation (OR=3.8; 95%CI=1.26-11.63; p=0.017) and female gender (OR=2.2; 95%CI=1.02-4.97; p=0.045) were independent predictors of better responses to quality of life, anxiety, and ICD acceptance, respectively.

## Discussion

Patient-reported outcomes are important health indicators, as they are reported from the individual’s own perspective regarding their illness and treatment, contributing to a patient-centered approach.^5,18^ Despite the safety that the ICD provides, individuals may progress with compromised quality of life, mainly as a result of shock therapies.^3,4,7^ Considering the need for a better understanding of the impact of the ICD, this study evaluated the quality of life, anxiety, and acceptance using the EQ-5D-3L, FSAS, and FPAS measuring instruments for the first time in Brazil.

Our study reflects data from real clinical practice in a tertiary cardiology hospital, mainly because it includes all patients operated on in a given period, regardless of the clinical profile, the procedure performed, and the type of ICD. We found a similar proportion of patients with ischemic heart disease (27.2%), non-ischemic heart disease (27.2%), and Chagas disease (23.1%), and there was a greater representation of reoperation procedures (54.4%).

The general health status results obtained by the visual analogue scale (EQ-VAS) in assessing the quality of life presented means ranging from 73.8 to 78.7, constituting similar values reported in the literature, which ranged from 62 .4 to 77.6, according to the study time and the ICD indication.^19,20^ The average scores obtained for the anxiety construct related to the ICD ranged from 23.5 to 23.9, denoting a mild anxiety level, similar to a cross-sectional study that reported average scores of 22.1 points.^8^ The scores obtained for the device acceptance were from 72.6 to 74.7 in 30 and 180 days, respectively, showing an adequate acceptance level and similar to previous studies, which reported scores ranging from 64.7 to 66.5.^8,21^

The rate of patients who achieved MID for the constructs quality of life, anxiety, and ICD acceptance was 22.4%, 24.5%, and 29.3%, respectively. The MID has been adopted in the literature because it reflects results that go beyond statistical significance values, enabling the detection of changes in the scores of measuring instruments that may represent significant changes from the patient’s point of view.^5,22^ A study that used the MID to classify individuals undergoing pacemaker implantation in relation to improvement in quality of life scores identified rates of 30% to 59% of patients who achieved MID.^23^ Although the pacemaker is a very similar device to the ICD, by acting on heart rate correction, it promotes a noticeable hemodynamic benefit, such that patients usually report improvements in their symptoms. On the other hand, patients with ICDs generally do not perceive any clinical benefit after implantation of the device and still live with the fear of receiving shock therapies.^3^

Notwithstanding that the study was not specifically designed to compare outcomes between different types of procedures, a similar proportion of patients achieving DMI was observed for the three analyzed constructs, both among individuals with a previously implanted ICD and those undergoing initial implantation. These findings suggest that the type of procedure, whether initial implants or reoperations, did not impact the study outcomes, in line with what has been already reported in the literature.^7-12^

Age greater than or equal to 60 years was considered a predictor of quality of life, increasing the chances of obtaining better scores by 2.5 times. Consistent with these results, patients with a mean age of 64.7 ± 9.4 years in a cohort study presented better quality of life scores.^24^ Acceptance of the device could mainly explain the better quality of life in this age group, as compared to young

individuals, since it has already been demonstrated that they have greater difficulties in accepting the ICD.^24,25^ On the other hand, age over 60 years has also been identified as a predictor for compromised quality of life, justified by the perception of a worse state of health, concerns, and changes in the lifestyles of patients in this age group.^11^

The absence of atrial fibrillation was identified as a predictor of better anxiety scores, increasing the chance of better responses by 3.8 times. Approximately 25% of patients with ICD have atrial fibrillation,^26^ and anxiety in these individuals could be explained by concern about palpitations and the use of anticoagulants due to the risk of adverse effects, such as bleeding and thromboembolism.^27^ Therefore, it is justifiable that patients in this study who had better anxiety scores were not diagnosed with atrial fibrillation.

Female sex was identified as a predictor of better ICD acceptance scores, increasing the chance of better responses by 2.2 times. Although high ICD acceptance levels have been reported in the female population,^28^ it has also been demonstrated that women may have difficulties accepting the device due to the influence on body image.^29^

Although this study enabled identifying predictors of better scores in PROs with ICDs, the generalization of the results is limited, mainly due to the fact that the sample size was reduced and that the data reflected the reality of a single institution. Conducting new studies with more representative samples and multicenter coverage may contribute to confirming the results, as well as identifying other predictors.

## Conclusion

The present study allowed us to better understand the profile of patients with ICDs in terms of quality of life, anxiety, and acceptance of the device and demonstrated the stability of the scores obtained between the two assessment moments. Identifying predictors for better quality of life scores, anxiety, and acceptance of the device can support implementing specific care for patients with a greater chance of presenting unfavorable results.
